# Supplementary material for: The Uptake of Heparanase into Mast Cells Is Regulated by Its Enzymatic Activity to Degrade Heparan Sulfate
Source: Int J Mol Sci. 2024 Jun 6;25(11):6281. doi: 10.3390/ijms25116281 (PMC11173065; doi:10.3390/ijms25116281)
Supplement: Supplementary file 1 [file ijms-25-06281-s001.zip › ijms-3025655-supplementary.pdf]

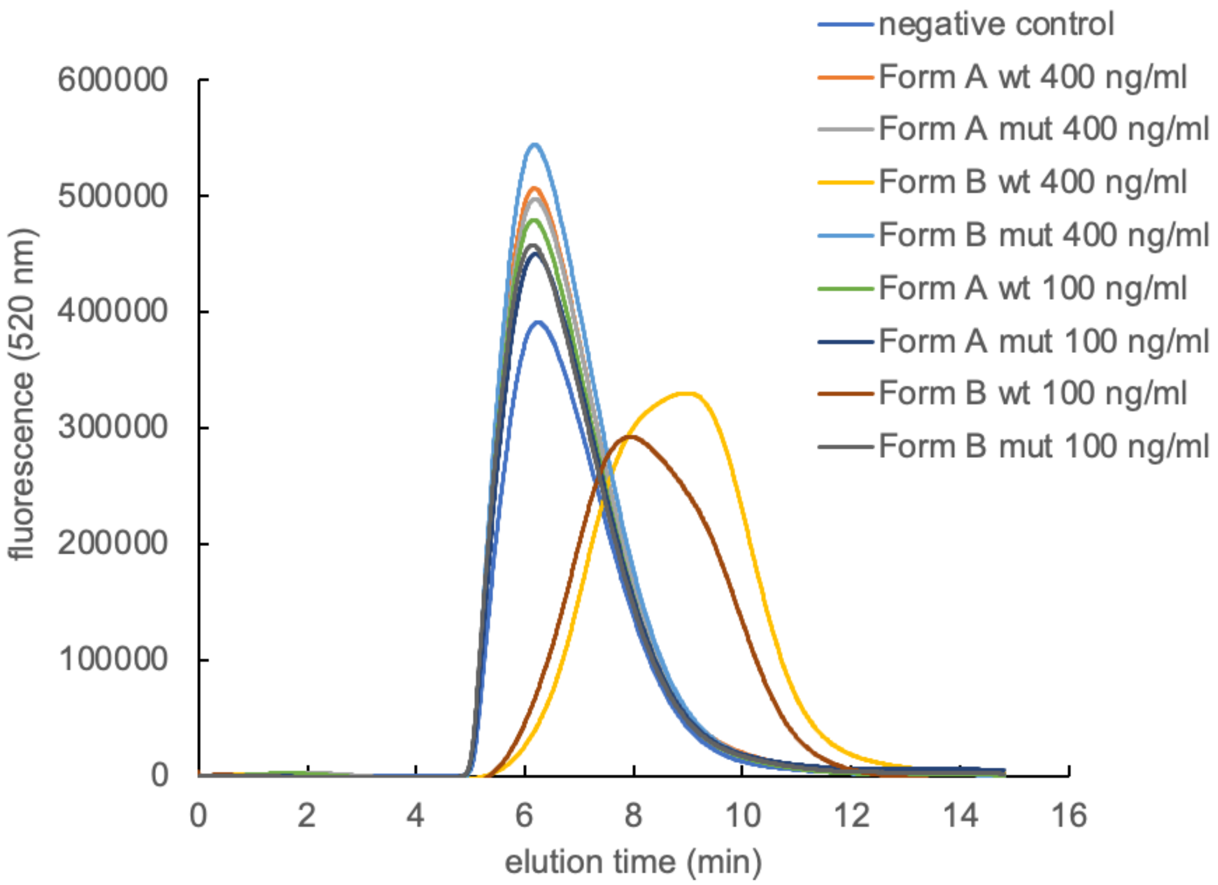

Figure S1.
Degradation of fluorescently-labeled HS by four types of Hpse proteins

Form A wt

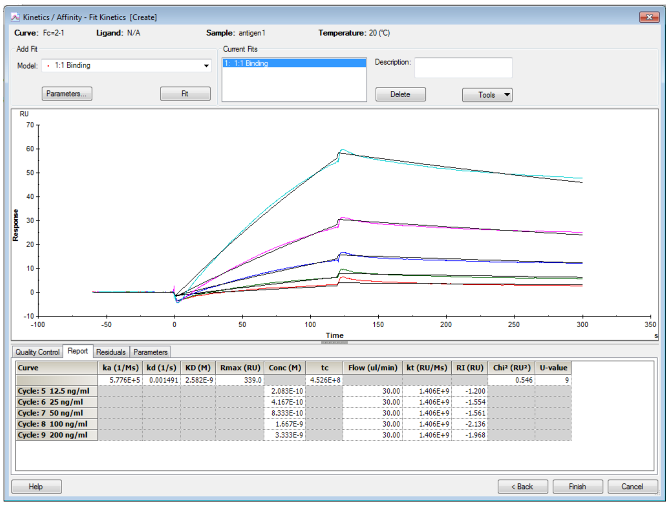

Form B wt

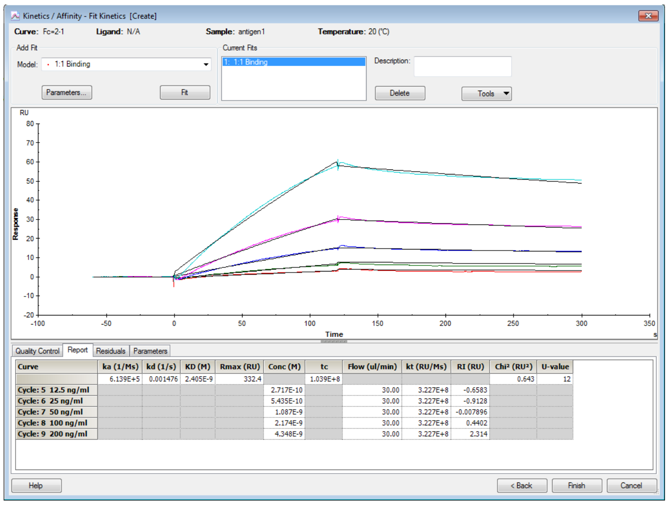

Form A mut

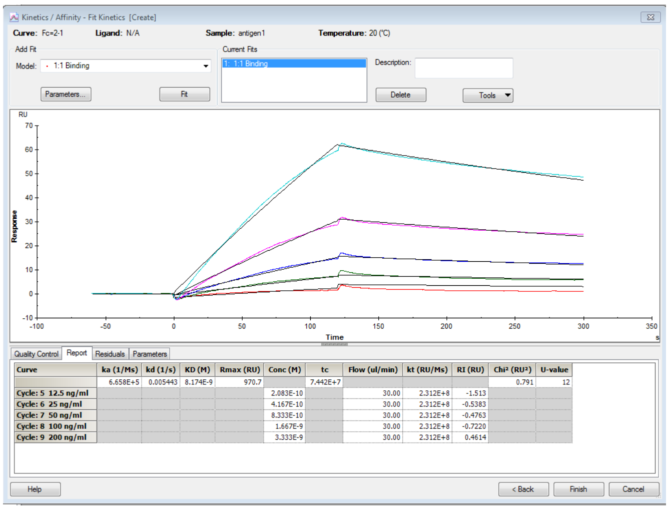

Form B mut

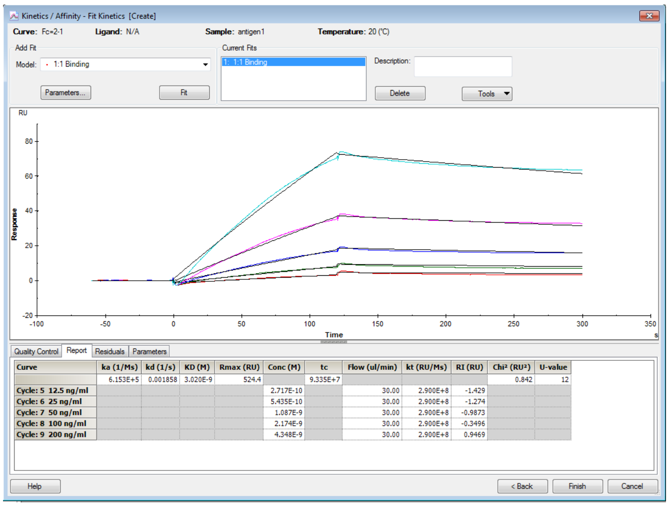

Figure S2.
Sensorgrams for the binding of four types of Hpse proteins to immobilized heparin

Table S1. Kinetic parameters for the interaction of four Hpse proteins with immobilized heparin.

	ka	kd	KD
L-Hpse wt	$(6.02 \pm 0.91) \times 10^{5}$	$(1.70 \pm 0.45) \times 10^{-3}$	$(3.00 \pm 1.19) \times 10^{-9}$
L-Hpse mut	$(6.23 \pm 1.36) \times 10^{5}$	$(3.37 \pm 1.47) \times 10^{-3}$	$(5.52 \pm 2.00) \times 10^{-9}$
M-Hpse wt	$(5.13 \pm 1.75) \times 10^{5}$	$(1.30 \pm 0.13) \times 10^{-3}$	$(2.88 \pm 1.05) \times 10^{-9}$
M-Hpse mut	$(6.42 \pm 0.45) \times 10^{5}$	$(2.73 \pm 0.97) \times 10^{-3}$	$(4.32 \pm 1.72) \times 10^{-9}$

Figure S1. Degradation of fluorescently-labeled HS by four types of Hpse proteins.

Fluorescently-labeled HS was mixed with four types of Hpse in 0.1 M acetic acid buffer (pH 5.5), and incubated for 20 hr at 37°C. HS degradation was detected using Superdex™ 75 Increase (5/150 GL, Cytiva, Uppsala, Sweden) as described elsewhere [54].

Figure S2. Sensorgrams for the binding of four types of Hpse proteins to immobilized heparin.

The interaction of four Hpse proteins with immobilized heparin was analyzed using a BIAcore T200 system as described previously [54]. The sensorgrams were overlayed with corresponding fitting curves generated by BIAevalution software 4.1 (Cytiva, Uppsala, Sweden) using a 1:1 binding model.

Table S1. Kinetic parameters for the interaction of four Hpse proteins with immobilized heparin.

The interaction of four Hpse proteins with immobilized heparin was analyzed using a BIAcore T200 system as described previously [54] with minor modification. The immobilization of biotinylated heparin was confirmed by the observation of a ~28 resonance unit increase on a streptavidin-immobilized sensor chip (BR-1005-31, Cytiva, Uppsala, Sweden). The binding reactions were carried out at 20°C. The recombinant Hpse proteins dissolved in running buffer (HBS-EP+: 10 mM HEPES, 0.15 M NaCl, 3 mM EDTA, and 0.05% (v/v) P20, pH 7.4) was injected onto the sensor chips at a flow rate of 30 μL/min. Association and dissociation periods were 120 s and 180 s, respectively. Kinetic parameters were evaluated with BIAevalution software 4.1 (Cytiva, Uppsala, Sweden) using a 1:1 binding model. Association and dissociation rate constants (ka and kd) as well as dissociation equilibrium constants (KD) were determined.
